# Gadoxetic acid-enhanced MRI combined with T1 mapping and clinical factors to predict Ki-67 expression of hepatocellular carcinoma

**DOI:** 10.3389/fonc.2023.1134646

**Published:** 2023-06-30

**Authors:** Ganbin Qiu, Jincan Chen, Weixiong Liao, Yonghui Liu, Zhongyan Wen, Yue Zhao

**Affiliations:** ^1^ Imaging Department of Zhaoqing Medical College, Zhaoqing, China; ^2^ Department of Radiology, The First People’s Hospital of Zhaoqing, Zhaoqing, China; ^3^ Department of Radiology, Central People’s Hospital of Zhanjiang, Zhanjiang, China; ^4^ Department of Radiology, The First Affiliated Hospital of Jinan University, Guangzhou, China

**Keywords:** hepatocellular carcinoma, Ki-67, magnetic resonance imaging, T1 mapping, nomogram

## Abstract

**Objectives:**

To explore the predictive value of gadoxetic acid-enhanced magnetic resonance imaging (MRI) combined with T1 mapping and clinical factors for Ki-67 expression in hepatocellular carcinoma (HCC).

**Methods:**

A retrospective study was conducted on 185 patients with pathologically confirmed solitary HCC from two institutions. All patients underwent preoperative T1 mapping on gadoxetic acid-enhanced MRI. Patients from institution I (n = 124) and institution II (n = 61) were respectively assigned to the training and validation sets. Univariable and multivariable analyses were performed to assess the correlation of clinico-radiological factors with Ki-67 labeling index (LI). Based on the significant factors, a predictive nomogram was developed and validated for Ki-67 LI. The performance of the nomogram was evaluated on the basis of its calibration, discrimination, and clinical utility.

**Results:**

Multivariable analysis showed that alpha-fetoprotein (AFP) levels > 20ng/mL, neutrophils to lymphocyte ratio > 2.25, non-smooth margin, tumor-to-liver signal intensity ratio in the hepatobiliary phase ≤ 0.6, and post-contrast T1 relaxation time > 705 msec were the independent predictors of Ki-67 LI. The nomogram based on these variables showed the best predictive performance with area under the receiver operator characteristic curve (AUROC) 0.899, area under the precision-recall curve (AUPRC) 0.946 and F1 score of 0.912; the respective values were 0.823, 0.879 and 0.857 in the validation set. The Kaplan–Meier curves illustrated that the cumulative recurrence probability at 2 years was significantly higher in patients with high Ki-67 LI than in those with low Ki-67 LI (39.6% [53/134] vs. 19.6% [10/51], *p* = 0.011).

**Conclusions:**

Gadoxetic acid-enhanced MRI combined with T1 mapping and several clinical factors can preoperatively predict Ki-67 LI with high accuracy, and thus enable risk stratification and personalized treatment of HCC patients.

## 1 Introduction

Hepatocellular carcinoma (HCC) is the third leading cause of cancer-related deaths worldwide (1). Although the prognosis of HCC patients has improved with advances in imaging and surgical techniques, the high rates of intrahepatic recurrence after surgical resection still remain a major challenge, and two thirds of the patients experience recurrence within 5 years (2, 3). Various factors affect intrahepatic recurrence, including microvascular invasion, degree of differentiation, satellite focus and related gene expression, and tumor cell proliferation (4).

The Ki-67 labeling index (LI) is an indicator of cell proliferation, which correlates to the biological behavior of tumors, treatment efficacy and prognosis (5, 6). Previous studies have shown that high Ki-67 LI is associated with poor overall survival (7–9) and recurrence-free survival (RFS) (9, 10). Currently, the Ki-67 LI of tumors is evaluated on the basis of postoperative immunohistochemical examination. Non-invasive estimation of the preoperative Ki-67 LI of HCC tissues may help predict patient prognosis and guide treatment decision-making. There are reports that texture analysis based on gadoxetic acid-enhanced magnetic resonance imaging (MRI) can preoperatively predict Ki-67 LI in HCC patients, and is superior to subjective MRI characteristics (11–13). In addition, radiomic score is also a reliable imaging biomarker of Ki-67 expression (14–18). However, both texture analysis and radiomics are not conducive to clinical application due to the complexity of the process, and the poor generalization and resolvability of the model (19, 20). T1 mapping is a non-invasive method for the quantification of T1 value in tissues. Moreover, it is directly proportional to the concentration of gadolinium contrast agent in tissues, and can reflect the uptake of gadoxetic acid more accurately (21). Some recent studies have shown that T1 mapping can be used to evaluate the degree of HCC differentiation (22, 23), histological grade of liver fibrosis (24, 25) and microvascular invasion (26). However, to our knowledge, the quantitative evaluation of Ki-67 LI in HCC using T1 mapping has not been well established.

Notably, the previous studies focused more on the image characteristics without identifying the importance of clinical characteristics on predictive performance. As we know, clinical characteristics such as biochemical and tumor biomarkers also played an important role in HCC diagnosis and prognosis (27–31). In addition, neutrophil-lymphocyte-ratio (NLR), platelet-lymphocyte-ratio (PLR), γ-glutamyl transpeptidase-lymphocyte ratio and other lab test data were found related to poor prognosis and had indications of therapeutic effects in HCCs (27, 29, 31). Accordingly, whether clinical characteristics can identify the Ki-67 LI in HCC remains unclear. The aim of this study was to investigate the predictive value of gadoxetic acid-enhanced MRI combined with T1 mapping and clinical indicators for preoperative Ki-67 LI in HCC.

## 2 Materials and methods

### 2.1 Study populations

**Figure 1:**
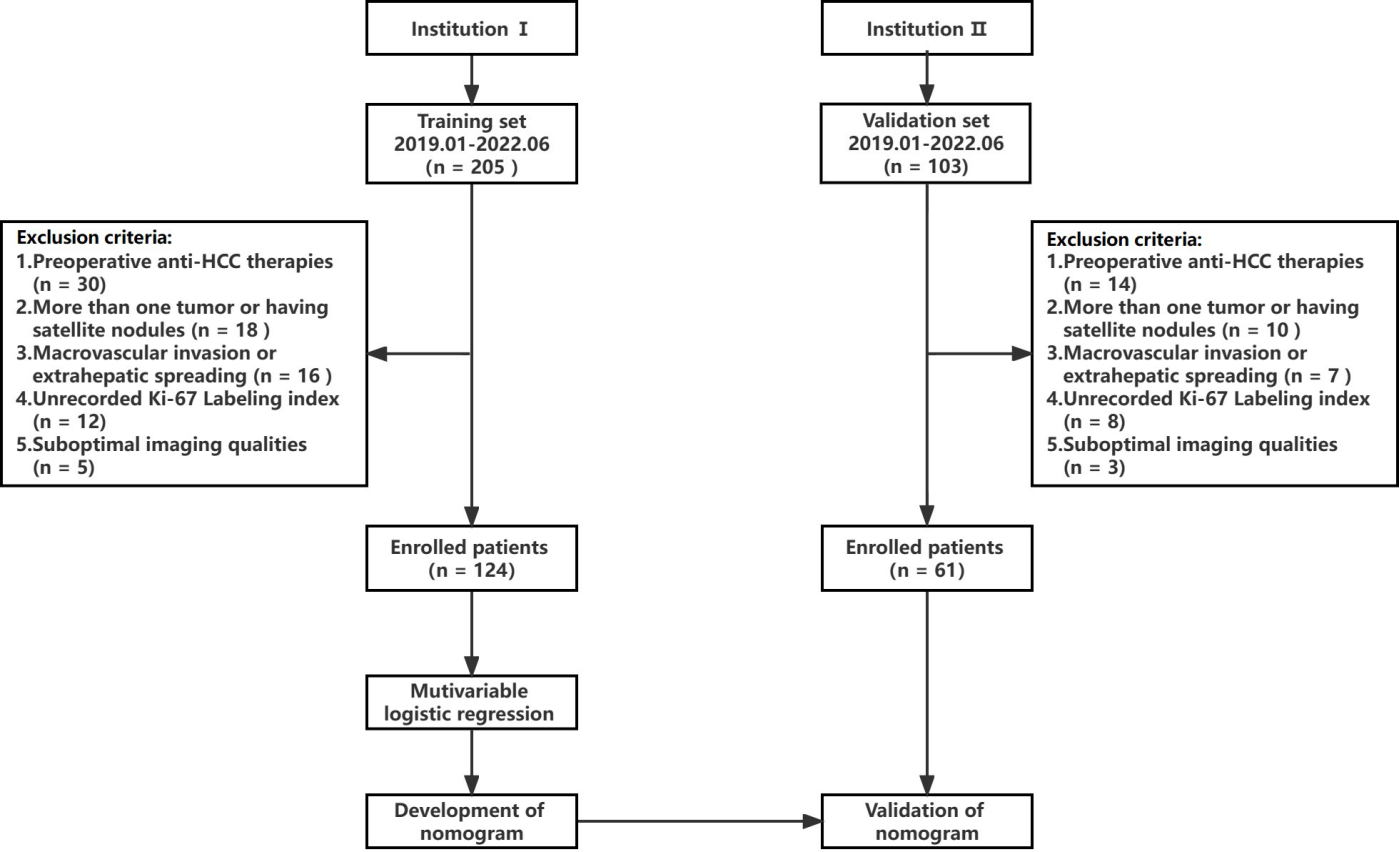
Study flowchart. HCC, hepatocellular carcinoma.

A retrospective study was conducted following approval by the hospital ethics committee, and patients were exempted from signing informed consent. The flow chart of data collection and research design is shown in **Figure 1**. The data of patients was retrieved from the First People’s Hospital of Zhaoqing (Institution I) and Central People’s Hospital of Zhanjiang (Institution II). The inclusion criteria for the patients were as follows (1): pathologically confirmed solitary HCC, (2) underwent gadoxetic acid-enhanced MRI within 2 weeks before surgery, including T1 mapping in the pre-enhanced and 20-minute hepatobiliary phase (HBP) after gadoxetic acid injection, and (3) availability of complete clinical and pathological data. The exclusion criteria were as follows: (1) alternative treatments such as radiofrequency ablation or transcatheter arterial chemoembolization (TACE) instead of resection surgery, (2) presence of more than one tumor or satellite nodules, (3) presence of macrovascular invasion or extrahepatic spreading, and (4) suboptimal MR image quality. The MR images of 124 patients from institution I were used as the training set to establish the predictive model for Ki-67 LI. The predictive performance of the model was evaluated on the 61 cases from institution II (validation set).

### 2.2 Clinicopathological analyses

Preoperative laboratory indicators included alpha-fetoprotein (AFP), alanine aminotransferase, aspartate aminotransferase, glutamyl transpeptidase, alkaline phosphatase, albumin, direct bilirubin, total bilirubin, serum creatinine, prothrombin time and international normalized ratio, neutrophil count, and platelet count. HCC was diagnosed on the basis of morphological criteria defined by the World Health Organization. The tumor tissue sections were immuno-stained using monoclonal mouse anti-human Ki-67 antibody (Beijing Zhongshan Golden Bridge Biotechnology Company, Beijing, China), and the Ki-67 LI was evaluated by calculating the frequency of Ki-67-positive cells. The samples with ≤10% positively stained cells were classified as Ki-67 LI^low^, and those with >10% positive cells as Ki-67 LI^high^ according to previous studies (11, 13, 32).

### 2.3 MRI protocol

MRI was performed at institution I and institution II using 3.0T (Magnetom Aera; Siemens Healthcare) and 3.0T (Magnetom Trio A Tim; Siemens Healthcare) MR scanners respectively. The scanning range covered the region from the top to the lower edge of the liver with an 8-channel phased-array coil as the receiver coil. Gadoxetic acid-enhanced MR images, including the pre-enhanced, enhanced arterial phase (AP, 20–40s), portal phase (PVP, 50–70s), transitional phase (TP, 100–120s), and 20 min HBP images were obtained. Gadoxetic acid (Primovist; Bayer Schering Pharma, Berlin, Germany) was injected into the cubital vein at the flow rate of 1 ml/s and the dose of 0.025 mmol/kg, and then flushed using 20 ml normal saline. A more detailed description of the MRI methods and specific sequences and parameters of MRI scans are shown in **Supplementary Materials 1.1** and **Table S1**.

### 2.4 Imaging analysis

Preoperative MRI images were retrospectively analyzed on the Picture Archiving and Communication System (PACS). The semantic and quantitative MRI features were evaluated by two abdominal radiologists independently (both with 8 years of experience in liver imaging, respectively) who were blinded to the clinical and pathological information. Discrepancies were resolved by consensus after reevaluating the images. Quantitative characteristics were obtained by averaging the two estimates. MRI features included tumor size, tumor margin, hemorrhage, necrosis, fat component, target sign, washout, rim arterial phase hyperenhancement, corona enhancement, intratumor arteries, radiologic capsule, and peritumoral hypointensity on HBP. All quantitative measurements were performed manually on the PACS. The region of interest (ROI) was placed as far as possible in the area with obvious enhancement of lesion to avoid necrosis, hemorrhage, fat, and artifacts. The area of ROI was about 1.0~1.5 cm^2^. The same lesion was measured three times with the same ROI, and the average was calculated. The signal intensity (SI) of the tumor and surrounding normal liver parenchyma were measured in the Pre, AP, PVP, EP and HBP images, and the tumor-to-liver contrast ratio (TLR), tumor enhancement index (TEI), relative tumor enhancement (RTE) and relative enhancement ratio (RER) were calculated. The pre-contrast and post-contrast T1 relaxation time were measured before and 20 minutes after the administration of the contrast medium (recorded as T1_Pre_ and T1_HBP_ respectively), and reduction rate of T1 relaxation time (ΔT1%) was calculated. The detailed description of semantic and quantitative MRI features is in the **Supplemental Materials 1.2**.

### 2.5 Follow-up

All patients were regularly followed up with imaging evaluation once every three months for the first two years after surgery. The recurrence was noted as new intrahepatic lesions and/or extrahepatic metastasis, with the following detailed criteria: 1) new intrahepatic lesions with typical imaging features of HCC, or confirmed by histopathology or with tumor staining during postoperative TACE; 2) extrahepatic metastasis confirmed by typical imaging features or histopathological analysis.

### 2.6 Statistical analysis

Student’s t-test was used to compare normally distributed continuous variables, while the Mann-Whitney U test was used for the non-normally distributed variables. The Chi-square test was used to compare binary categorical variables. The interclass correlation coefficient (ICC) of quantitative data between the two observers was calculated. To identify the independent predictors for high Ki-67 LI without multicollinearity (variance inflation factor, VIF < 5), multivariate backward logistic regression analysis was performed. Receiver operating characteristic (ROC) curves were plotted to determine the cut-off values of the continuous variables for predicting Ki-67 LI by calculating the maximum Youden index. The area under the ROC curve (AUROC) was calculated to evaluate the predictive performance, and DeLong’s test was used to compare the AUROC values between two models. Due to the imbalance between the data of Ki-67 LI^low^ and Ki-67 LI^high^ patients, the F1 score and the area under the precision-recall curve (AUPRC) were also calculated to compare the predictive performances. The discriminatory abilities of the models were quantified by net reclassification index (NRI), where NRI > 0 indicates positive improvement of the new model over the old one. The calibration curves of the nomogram were assessed by the Hosmer-Lemeshow test for consistency. Decision curve analysis (DCA) was performed to evaluate the clinical utility of the nomogram by quantifying the net benefit under different threshold probabilities. All statistical analyses were performed using the SPSS software (version 23.0, IBM Corp.) and R software (version 4.1.3, http://www.r-project.org). A *p*-value < 0.05 was considered statistically significant.

## 3 Results

### 3.1 Clinicopathological features of the training and validation sets

A total of 124 patients from institution I were included in this study, of which 35 had low Ki-67 LI and 89 had high Ki-67 LI. Furthermore, 61 patients were included from institution II, and the Ki-67 LI was respectively low and high in 16 and 45 patients. The clinicopathological features of the training and validation sets were similar (**Table 1**). Univariate analysis of clinical factors in the training set showed that ALT (*p*=0.043), NLR (*p*=0.036), PLR (*p*=0.045) and AFP (*p*=0.024) were significantly associated with Ki-67 LI (**Supplementary Table S2**), and thus incorporated into the logistic regression model. The results indicated that AFP >20 ng/mL (*p*<0.001, OR=6.764, 95% CI: 2.225-23.792) and NLR >2.25 (*p*=0.038, OR=3.527, 95% CI: 1.023-16.274) were independent factors of high Ki-67 LI.

**Table 1:** Baseline clinical characteristics of the training and validation sets.

Characteristic	Total (n = 185)	Training set (n = 124)	Validation set (n = 61)	p value
Age (years)	45 [42, 67]	56 [50, 68]	56 [45, 65]	0.264
Sex (male)	168 (90.8%)	110 (88.7%)	58 (95.1%)	0.511
HBsAg				0.081
Negative	30 (16.2%)	17(13.7%)	13 (21.3%)	
Positive	155 (83.8%)	107 (86.3%)	48 (78.7%)	
ALT (U/L)	31.00 [22.50, 51.00]	36.00 [21.50, 57.35]	32.50 [22.50, 54.00]	0.677
AST (U/L)	40.50 [24.50, 45.55]	40.00 [24.00, 46.50]	42.50 [22.00, 55.75]	0.846
GGT (U/L)	55.00 [38.50, 108.65]	52.00 [35.50, 106.25]	53.70 [32.45, 125.50]	0.613
ALP (U/L)	82.50 [68.55, 102.50]	81.00 [70.00, 106.00]	85.00 [67.50, 108.55]	0.327
ALB (g/L)	40.35 [35.30, 43.47]	38.80 [37.58, 42.25]	36.50 [35.50, 42.70]	0.162
TBIL (µmol/L)	14.62 [12.00, 16.32]	13.62 [10.34, 16.56]	14.50 [12.60, 18.61]	0.665
SCr (U/L)	75.20 [66.15, 87.50]	75.60 [67.00, 86.85]	76.00 [68.28, 87.30]	0.548
PT (s)	11.60 [11.40, 12.50]	11.80 [11.60, 12.60]	11.95 [11.40, 12.58]	0.756
INR				0.673
≤1.0	76 (41.1%)	49 (39.5%)	27 (44.3%)	
>1.0	109 (58.9%)	75 (60.5%)	34 (55.7%)	
NLR	2.01 [1.44, 3.45]	2.43 [1.83, 3.42]	2.05 [1.58, 3.72]	0.403
PLR	103.45 [70.55, 148.65]	112.96 [83.15, 144.27]	102.83 [71.30, 156.22]	0.761
AFP (ng/mL)	24.35 [4.24, 132.41]	18.55 [7.39, 92.32]	28.53 [5.53, 545.00]	0.206

Continuous variables are presented as median [inter-quartile range, IQR]. Categorial variables are presented as number (percentage). p-values represent the result of comparison of the training set with the test set.

HBsAg, hepatitis B surface antigen; ALT, alanine aminotransferase; AST, aspartate aminotransferase; GGT, glutamyl transpeptidase; ALP, alkaline phosphatase; ALB, albumin; TBIL, total bilirubin; SCr, serum creatinine; PT, prothrombin time; INR, international normalized ratio; NLR, neutrophil to Lymphocyte ratio; PLR, platelet to Lymphocyte ratio; AFP, alpha fetoprotein.

### 3.2 MRI features of HCCs related to Ki-67 LI

Univariable analysis of semantic features in the training set showed that non-smooth tumor margin (*p*<0.001), hemorrhage (*p*=0.031) and necrosis (*p*=0.035) were more frequent in the Ki-67 LI^high^ group compared to the Ki-67 LI^low^ group (**Supplementary Table S3**). Furthermore, the tumor size, TLR_TP_, TLR_HBP_, TEI_HBP_, T1_Pre_ and T1_HBP_ were also significantly different between the two groups (**Table 2**), of which T1_HBP_ showed the best predictive performance for high Ki-67 LI, with an AUROC of 0.726, and sensitivity and specificity of 73.25% and 71.69% respectively (**Supplementary Table S4**). Furthermore, the ICC values of quantitative features in the training and validation sets were all above 0.75 (0.76 ~ 0.89; (**Supplementary Table S5**), indicating that the two radiologists were consistent in their analysis.

**Table 2:** Comparison of quantitative MRI parameters between Ki-67 LI^low^ and Ki-67 LI^high^ groups in the training set.

	Low Ki-67 LI (n = 35)	High Ki-67 LI (n = 89)	p value	ICC
Tumor size (cm)	3.15 [2.26, 4.36]	5.24 [2.62, 5.51]	0.002*	0.88
TLR_AP_	1.33 ± 0.25	1.38 ± 0.42	0.667	0.82
TEI_AP_	1.63 ± 0.31	1.73 ± 0.42	0.632	0.85
RTE_AP_	0.72 ± 0.41	0.80 ± 0.43	0.914	0.76
RER_AP_	0.84 [0.35, 1.05]	0.72 [0.42, 1.02]	0.445	0.80
TLR_PVP_	0.78 [0.83, 1.24]	0.84 [0.71, 1.13]	0.265	0.84
TEI_PVP_	1.23 [1.03, 1.42]	1.13 [0.95, 1.40]	0.232	0.77
RTE_PVP_	0.85 [0.52, 1.14]	0.70 [0.33, 1.11]	0.244	0.84
RER_PVP_	1.42 [0.89, 2.46]	1.04 [0.64, 1.84]	0.065	0.76
TLR_TP_	0.89 [0.78, 1.07]	0.78 [0.65, 0.95]	0.025*	0.83
TEI_TP_	1.12 ± 0.23	1.17 ± 0.34	0.558	0.82
RTE_TP_	0.74 ± 0.31	0.72 ± 0.36	0.830	0.80
RER_TP_	1.11 [0.75, 1.43]	0.95 [0.62, 1.23]	0.065	0.79
TLR_HBP_	0.67 ± 0.14	0.53 ± 0.16	0.004*	0.86
TEI_HBP_	0.78 ± 0.12	0.65 ± 0.15	0.026*	0.85
RTE_HBP_	0.37 [0.23, 0.54]	0.26 [0.14, 0.58]	0.314	0.87
RER_HBP_	0.45 [0.26, 0.68]	0.37 [0.24, 0.56]	0.058	0.81
T1_Pre_ (msec)	1243.07 ± 257.41	1385.36 ± 239.28	0.047*	0.83
T1_HBP_ (msec)	744.17 ± 162.74	845.21 ± 156.42	0.007*	0.82
ΔT1%	0.41 ± 0.14	0.38 ± 0.13	0.247	0.78

*p<0.05. Continuous variables are presented as median [inter-quartile range, IQR] or mean ± standard deviation. Categorial variables are presented as number (percentage).

AP, arterial phase; PVP, portal venous phase; TP, transition phase; HBP, hepatobiliary Phase; TLR, tumor to liver contrast ratio; TEI, tumor enhancement index; RTE, relative tumor enhancement; RER, relative enhancement ratio; Pre, pre-enhancement; T1_Pre_: pre-contrast T1 relaxation time; T1_HBP_, T1 relaxation time in the hepatobiliary phase; ΔT1%, reduction rate of T1 relaxation time; ICC, intraclass correlation coefficient.

### 3.3 Development and validation of predictive models for Ki-67 LI

**Figure 2:**
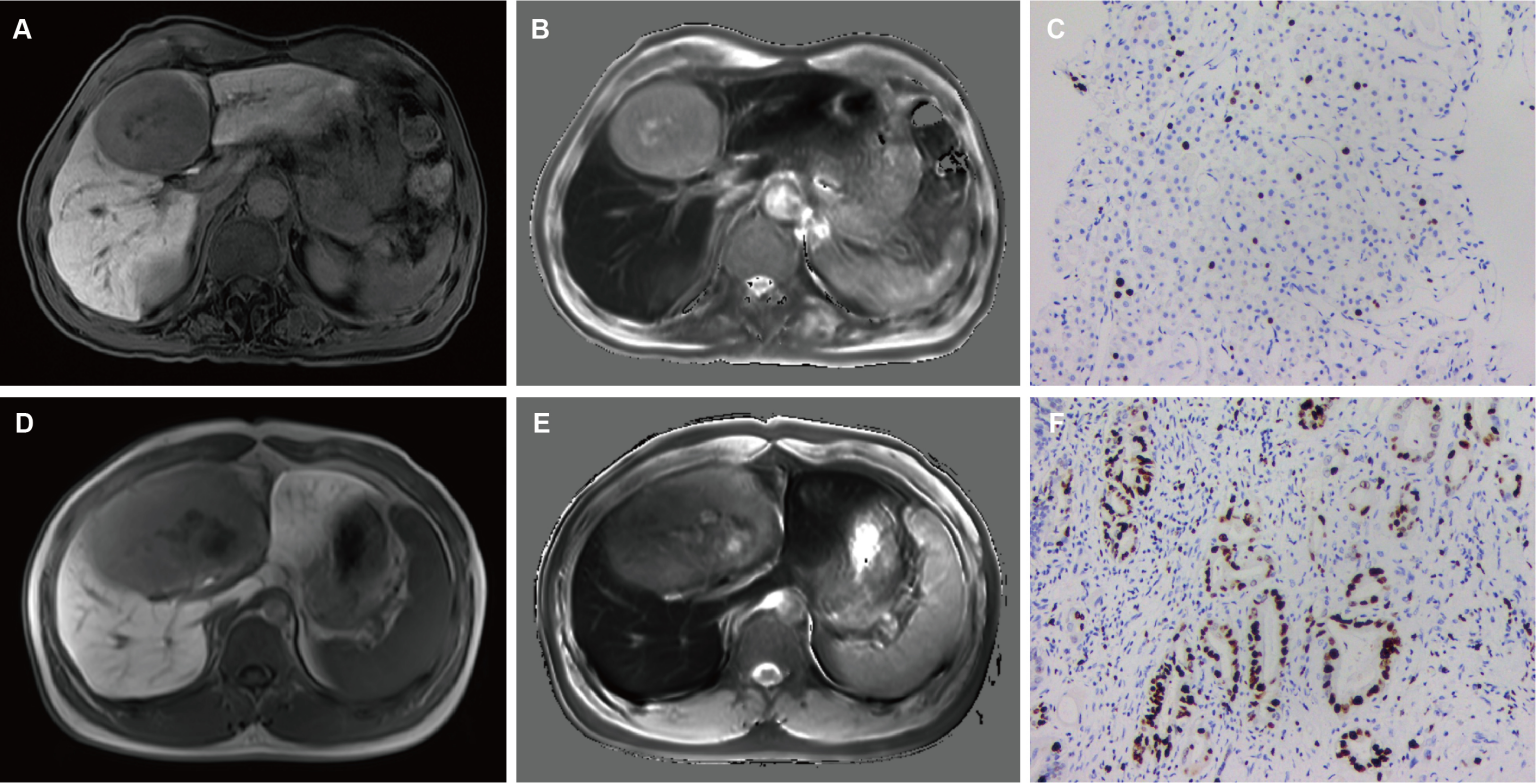
A 49-year-old male with mildly elevated AFP (36 ng/mL) and NLR 1.87. Smooth tumor margin and TLR_HBP_ was 0.68 **(A)**. T1_HBP_ was 682 ms **(B)**. The tumor had low Ki-67 LI (10%, IHC ×400) **(C)**. A 65-year-old male with elevated AFP (124 ng/mL) and NLR 2.35. Non-smooth tumor margin and TLR_HBP_ was 0.55 **(A)**. T1_HBP_ was 786 ms **(B)**. The tumor had high Ki-67 LI (40%, IHC ×400) **(C)**. AFP, alpha-fetoprotein; NLR, neutrophils to lymphocyte ratio; TLR_HBP_, tumor-to-liver contrast ratio in the hepatobiliary phase; T1_HBP_, T1 relaxation time on the hepatobiliary phase; LI, labeling index; IHC, immunohistochemistry.

After excluding SI-based quantitative parameters with high collinearity (VIF > 5), TLR_HBP_ with the highest Youden index was selected for constructing the predictive model. Multivariable logistic regression showed that AFP > 20ng/mL (*p*=0.022, OR=3.863, 95%CI: 1.218 ~12.245), NLR > 2.25 (*p*=0.005, OR=9.159, 95%CI: 1.962 ~ 42.753), non-smooth tumor margin (*p*=0.013, OR=4.776, 95%CI: 1.393 ~ 16.374), TLR_HBP_ ≤ 0.6 (*p*=0.003, OR=6.993, 95%CI: 1.962 ~ 24.927) and T1_HBP_ >705 msec (*p*<0.001, OR=10.673, 95%CI: 2.614 ~ 43.583) were independent predictors of high Ki-67 LI in HCC tumors (**Table 3**, **Figure 2**).

**Table 3:** Univariable and multivariable logistic regression analysis for predictors of Ki-67 LI in the training set.

Characteristics	Univariable		Multivariable	
	OR (95% CI)	p value	OR (95% CI)	p value
AFP > 20 ng/mL	5.977 (2.484 ~ 14.383)	<0.001	3.863 (1.218 ~ 12.245)	0.022
ALT > 40 U/L	2.763 (1.193 ~ 6.399)	0.018		
NLR > 2.25	3.133 (1.180 ~ 8.318)	0.022	9.159 (1.962 ~ 42.753)	0.005
PLR > 138.5	4.031 (1.234 ~ 13.172)	0.021		
Tumor size > 5 cm	3.911 (1.548 ~ 9.879)	0.004		
Non-smooth tumor margin	5.526 (2.373 ~ 12.868)	<0.001	4.776 (1.393 ~ 16.374)	0.013
Necrosis	2.444 (1.052 ~ 5.679)	0.038		
Hemorrhage	3.488 (1.068 ~ 11.398)	0.039		
TLR_HBP_ ≤ 0.6	6.645 (2.809 ~ 15.717)	<0.001	6.993 (1.962 ~ 24.927)	0.003
T1_Pre_ > 1280 msec	3.268 (1.452 ~ 7.352)	0.004		
T1_HBP_ > 705 msec	4.644 (2.017 ~ 10.695)	<0.001	10.673 (2.614 ~ 43.583)	<0.001

AFP, alpha-fetoprotein; ALT, alanine aminotransferase; NLR, neutrophil to lymphocyte ratio; PLR, platelet to lymphocyte ratio; TLR_HBP_, tumor-to-liver signal intensity ratio in the hepatobiliary phase; T1_Pre_: pre-contrast T1 relaxation time; T1_HBP_, T1 relaxation time in the hepatobiliary phase; OR, odd ratio; CI, confidence interval.

**Figure 3:**
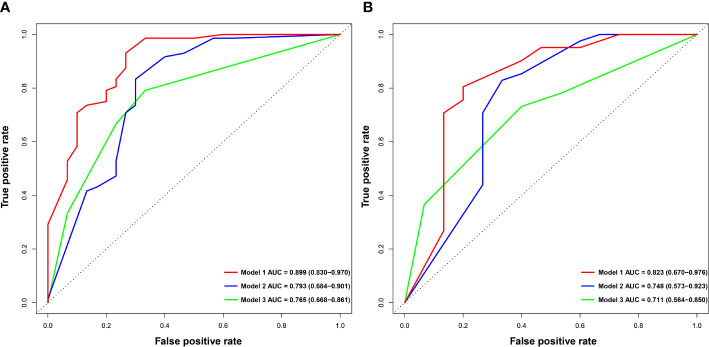
Comparison of ROC curves for predicting Ki-67 LI in HCC. ROC curves of combination model (Model 1), imaging model (Model 2) and clinical model (Model 3) in the training **(A)** and validation **(B)** sets. ROC, receive operating characteristics.

We constructed models based on clinical data, imaging data and combined data. The combination model showed better diagnostic performance compared to the clinical and imaging models in both training and validation sets. The AUROC of the combined model in the training and validation sets was 0.899 and 0.823 respectively, compared to 0.792 and 0.748 for the imaging model, and 0.765 and 0.711 for the clinical model (**Table 4**, **Figure 3**). The diagnostic performance of the combined model had significantly improved relative to the clinical model (*p*=0.008) and the imaging model (*p*=0.035) according to DeLong’s tests, whereas the imaging model had no significant difference compared to the clinical model (*p*=0.714). Precision-recall curve also showed that the combination model had the largest AUPRC (0.946) and F1 score (0.912) in the training set, and the respective values in the external validation set were 0.879 and 0.857. Compared to the clinical model and imaging model, the NRIs of the combined model were 21% (*p*=0.023) and 13% (*p*=0.038) in the training set, and 27% (*p*=0.018) and 11% (*p*=0.046) in the validation set respectively, indicating that the combined model had better efficacy.

**Table 4:** Comparison of the imaging, clinical and combined models.

Models	Data sets	ACC	SEN	SPE	PPV	NPV	AUROC	AUPRC	F1 Score
Clinical	Training	0.755	0.792	0.667	0.851	0.571	0.765	0.877	0.820
	Validation	0.696	0.732	0.600	0.833	0.450	0.711	0.836	0.779
Imaging	Training	0.794	0.833	0.700	0.869	0.636	0.793	0.885	0.851
	Validation	0.786	0.829	0.667	0.872	0.588	0.748	0.875	0.849
Combined	Training	0.873	0.931	0.733	0.893	0.815	0.899	0.946	0.912
	Validation	0.804	0.805	0.800	0.917	0.600	0.823	0.879	0.857

ACC, accuracy; SEN, sensitivity; SPE, specificity; PPV, positive predictive value; NPV, negative predictive value; AUROC, the area under the receiver operator characteristic curve; AUPRC, the area under the precision-recall curve.

**Figure 4:**
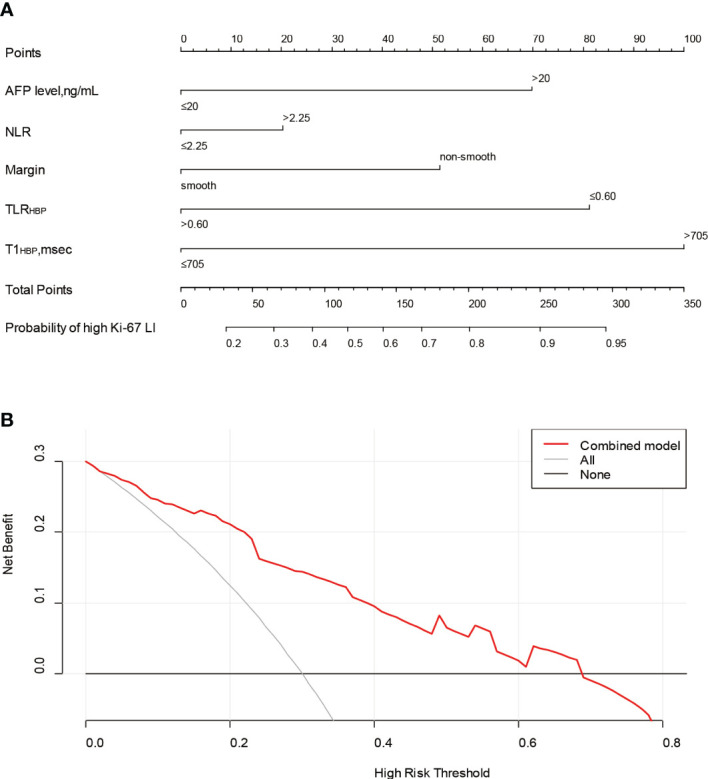
The nomogram and decision curve to predict Ki-67 LI in HCC. The nomogram **(A)** was developed based on the combination model. Predictor points are shown on the uppermost point scale that corresponds to each variable. The points for all variables are added in the bottom scale and translated into the probability of high Ki-67 LI. Decision curve **(B)** analysis of the prediction model for external validation set. The X-axis is the probability threshold. Y-axis represents the net benefit, which is calculated by gaining true positives and deleting false positives. AFP, alpha-fetoprotein; NLR, neutrophils to lymphocyte ratio; TLR_HBP_, tumor-to-liver contrast ratio in the hepatobiliary phase; T1_HBP_, T1 relaxation time on the hepatobiliary phase; LI, labeling index.

**Figure 5:**
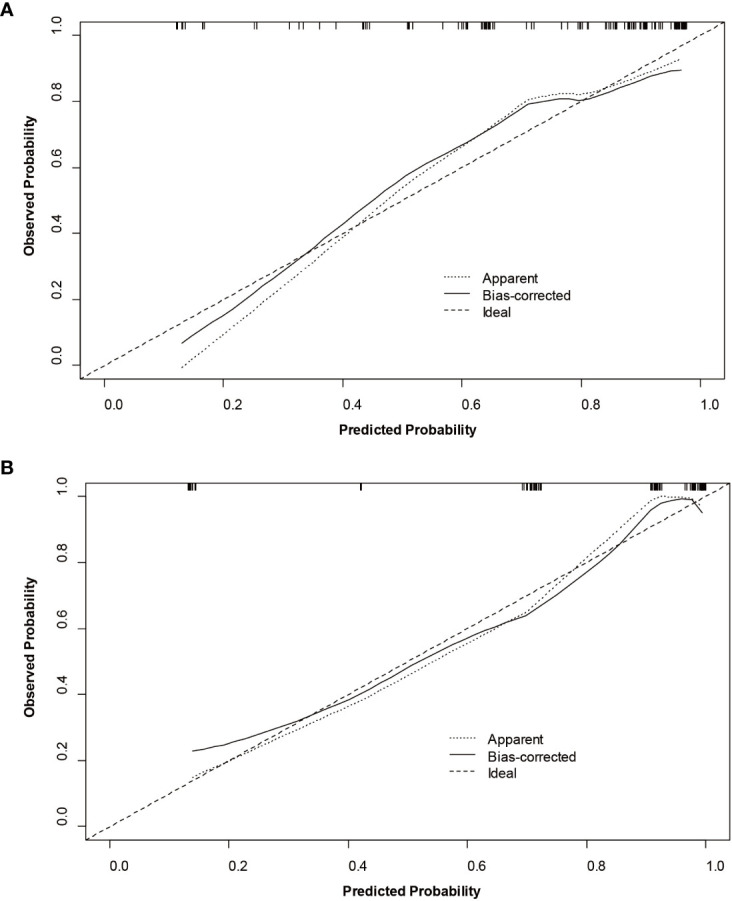
Calibration curves of the nomogram for the training **(A)** and validation **(B)** sets to predict Ki-67 LI in HCC.

The nomogram and decision curves revealed substantial clinical benefit of the combined model in predicting HCC with high Ki-67 LI (**Figure 4**). The DCAs for the clinical, imaging, and combined models in the training set are shown in **Supplementary Figure S1**. The calibration curves showed good agreement between predicted and observed probabilities of HCC with high Ki-67 LI in both the training (*p*=0.582) and validation (*p*=0.265) sets (**Figure 5**).

### 3.4 Early recurrence after hepatectomy

**Figure 6:**
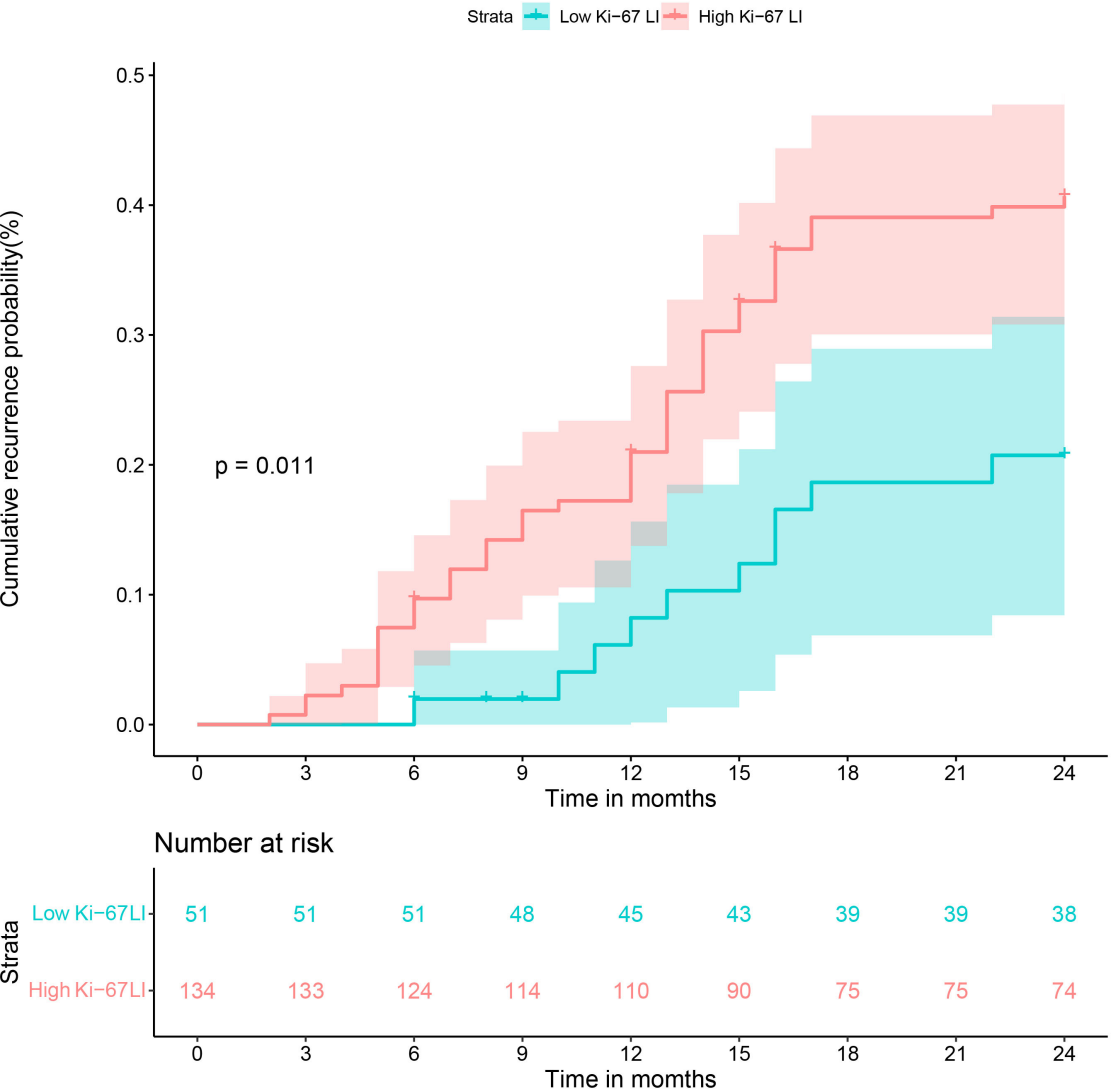
Cumulative recurrence curves of HCC patients with low and high Ki-67 LI after curative resection.

All patients had completed the early recurrence follow-up, and recurrence data was available for 63 patients within two years after hepatectomy. The Kaplan–Meier curves illustrated that the cumulative recurrence probability at 2 years was significantly higher in patients with high Ki-67 LI than in those with low Ki-67 LI (39.6% [53/134] vs. 19.6% [10/51], *p* = 0.011; **Figure 6**).

## 4 Discussion

We successfully developed and validated a nomogram to predict Ki-67 LI in HCC based on gadoxetic acid-enhanced MRI combined with T1 mapping and clinical indicators. The model demonstrated good predictive efficiency and clinical utility, and can facilitate personalized risk stratification and treatment decision-making for patients with HCC.

TLR_HBP_ was identified as an independent factor of high Ki-67 expression in HCC, which is consistent with previous studies (32, 33). The quantitative parameters, including TLR_HBP_, TEI_HBP_ and TLR_TP_, were based on the SI from either HBP or TP, which may better represent the Ki-67 LI of HCC given the rationale of gadoxetic acid (32). Among these quantitative parameters derived from either HBP or TP, TLR_HBP_ had the highest diagnostic performance for Ki-67 LI. The reason why HCCs with higher Ki-67 LI tend to demonstrate lower relative tumoral SI probably is that normal hepatocytes gradually turn into actively proliferated and uncontrolled malignant tumor cells with higher Ki-67 LI during multistep hepatocarcinogenesis, while at the same time, the expression of organic anion transporting polypeptide (OATP) usually decreased, hence resulting in less uptake of gadoxetic acid (34, 35). However, the correlation between Ki-67 LI and OATP may need to be investigated further. T1_HBP_ derived from T1 mapping showed the best diagnostic performance among all quantitative parameters, possibly due to the fact that T1 relaxation time is inversely proportional to the concentration of gadolinium contrast agent. As more gadolinium enters the tumor tissue in the HBP, it effectively shortens the T1 relaxation time. Furthermore, T1 relaxation time is an absolute value, which is not affected by scanning sequence parameters (26). However, SI is a relative value that is affected by technical factors and does not have a linear relationship with the concentration of the contrast agent. Thus, T1 relaxation time is more accurate and reliable than SI.

In addition to TLR_HBP_ and T1_HBP_, other independent predictors of high Ki-67 LI included serum AFP levels, NLR, and non-smooth margin. AFP is a marker of HCC and is associated with Ki-67 LI. Elevated serum AFP in HCC patients is correlated to poor differentiation, microvascular invasion and tumor recurrence (34, 36), which is consistent with the biological behavior of HCCs with high Ki-67 LI. Furthermore, Besides, our study found that NLR was an independent factor of high Ki-67 expression in HCC. The possible resolution is that changes in NLR affect the levels of some proinflammatory mediators associated with oncogenic effects, thus accelerating tumor cell proliferation and invasion (37). Although there is epidemiological evidence that inflammation is a risk factor for many human cancers (36, 37), the underlying mechanisms are unclear. Finally, non-smooth tumor margins, necrosis, and hemorrhage were more common in the Ki-67 LI^high^ group, which is consistent with previous studies (12, 13, 38).

We constructed predictive models for Ki-67 LI based on clinical, imaging and combined data, and found that the combined model had the best predictive performance. This suggests that combining clinical information and imaging features can provide complementary information and improve predictive performance. Some studies have established models based on texture analysis (11–13) or radiomics (14–16), which have also achieved good results in predicting Ki-67 LI in HCC. However, obscure algorithms and complicated operations limit their clinical applications. In addition, most of these studies were conducted on single-center data without an independent external validation set, thus limiting the generalizability of the results. An external dataset is necessary to ensure clinical translatability of such models. In our study, the combination model showed robust performance in the external validation set with different MRI scanners and parameter settings.

There are some limitations in our study that ought to be considered. First, the retrospective design of the study may have introduced selection bias. We focused on single resectable HCC tumors due to the difficulty in obtaining surgical specimens of multiple lesions and those with macrovascular invasion, which limits the extrapolation of our results to different populations. Thus, future studies should explore the correlation between MRI features and Ki-67 expression in multiple tumors. Second, the small sample size may affect the robustness of this model, which will have to be further optimized through large-scale and multicenter studies. Third, we did not calculate the T1 relaxation time ratio of tumor to liver parenchyma since the presence of liver cirrhosis might have influenced the results.

## 5 Conclusions

Gadoxetic acid-enhanced MRI combined with T1 mapping and several clinical factors can preoperatively predict the Ki-67 LI of HCC, and therefore guide treatment and prognostic assessment. Nevertheless, the clinical utility of our prediction model in combination with clinico-radiological features will have to be validated in future randomized trials to guide individualized therapy.

## Data availability statement

The raw data supporting the conclusions of this article will be made available by the authors, without undue reservation.

## Ethics statement

The studies involving human participants were reviewed and approved by The hospital ethics committee of the first people’s hospital of Zhaoqing. Written informed consent for participation was not required for this study in accordance with the national legislation and the institutional requirements.

## Author contributions

GQ, JC, and WL conducted the literature search. GQ, WL, YL, and YZ designed the study. GQ, JC, WL, and YL collected the data. GQ, JC, WL, YL, and YZ analyzed the data. All authors verified the data. GQ, JC, WL, YL, and YZ edited the manuscript. WL, YL, and YZ reviewed the manuscript. All authors contributed to the article and approved the submitted version.
