# Supplementary material for: Gadoxetic acid-enhanced MRI combined with T1 mapping and clinical factors to predict Ki-67 expression of hepatocellular carcinoma
Source: Front Oncol. 2023 Jun 30;13:1134646. doi: 10.3389/fonc.2023.1134646 (PMC10348748; doi:10.3389/fonc.2023.1134646)

**Supplementary Material**

**1. Supplementary Methods**

**1.1 MRI protocols of the two Institutions**

All study patients from institutions I and II underwent gadoxetic acid-enhanced MRI using 3.0-T or 1.5-T systems. Technical details of MRI protocols at the two institutions are outlined in Table S1.

**Table S1** MR scan sequence and parameters.

| Acquisition Sequence | Matrix Size | Section Thickness (mm) | Intersection Gap (mm) | Repetition Time  (msec) | Echo Time  (msec) | Flip Angle (º) |
| --- | --- | --- | --- | --- | --- | --- |
| Magneton Trio A Tim 3.0-T system (Siemens Healthcare, Erlangen, Germany) | | | | | | |
| T_1_WI-VIBE | 320×240 | 3 | 1 | 4.00 | 1.29/2.52 | 9 |
| T_1_WI-TWIST-VIBE | 288×216 | 3 | 0 | 3.89 | 1.23/ 2.46 | 13 |
| T_2_WI-HASTE | 320×240 | 3 | 1 | 3637 | 87 | 140 |
| DWI (b=50,800 sec/mm^2^) | 128×128 | 5 | 1 | 6200 | 50 | / |
| T_1_ mapping | 224×168 | 4 | 1 | 5.01 | 2.3 | (3, 15) |
| Magneton Skyra 3.0-T system (Siemens Healthcare, Erlangen, Germany) | | | | | | |
| T_1_WI-VIBE | 192×256 | 5 | 1 | 4.00 | 1.31/2.55 | 10 |
| T_1_WI-TWIST-VIBE | 320×208 | 3 | 0 | 3.36 | 1.25/ 2.26 | 12 |
| T_2_W1-HASTE | 320×224 | 5 | 1 | 2000 | 81 | 150 |
| DWI (b=50,800 sec/mm^2^) | 192×154 | 5 | 1 | 5100 | 73 | / |
| T_1_ mapping | 256×204 | 3 | 1 | 3.37 | 1.18 | (2, 11) |

Abbreviation: VIBE, volumetric interpolated breath-hold examination; TWIST, time-resolved imaging with interleaved stochastic trajectories; HASTE, half-Fourier acquisition single-shot turbo spin-echo; DWI, diffusion-weighted imaging; GRE, gradient recalled echo.

**1.2 Image Analysis**

The semantic MRI features included:

1. **Tumor margin**: categorized as smooth margin and non-smooth margin on hepatobiliary phase (HBP) images, round or oval tumors with smooth contour were identified as smooth margin, while non-smooth tumor margins included local protruding type, polynodular fusion type and irregular infiltrating type;
2. **Hemorrhage**: high signal intensity (SI) on T_1_-weighted imaging (T_1_WI) with variable SI on T_2_-weighted imaging (T_2_WI);
3. **Necrosis**: defined as high SI areas inside the lesion on T_2_WI or low SI areas not enhanced during enhancement;
4. **Fat component**: compared with the in-phase images, the tumor area had decreased SI on out-phase images;
5. **Target signs**: ring-like high SI with central relative hypointensity on diffusion-weighted imaging (DWI); Moderate to marked hypointensity in the periphery and mild hypointensity in the central area on HBP;
6. **Wash out**: The enhancement of lesions in the arterial phase (AP) was reduced and lower than that in the peripheral liver parenchyma in the portal phase (PVP) or transition phase (TP);
7. **Peritumoral enhancement**: irregular and patchy peritumoral hyperenhancement in the AP that became isointense with normal liver parenchyma in the PVP or TP;
8. **Rim Arterial phase hyperenhancement (rim APHE)**: ring-like enhancement with central relative hypointensity;
9. **Intratumor arteries**: visible intratumor blood vessel on the AP;
10. **Radiographic capsule**: smooth, uniform, sharp border around most or all of the tumor, visible as an enhancing rim in the PVP or AP, and classified as capsular (complete or incomplete capsule) or non-capsular;
11. **Peritumoral hypointensity on HBP**: irregular or wedge-shaped hypointense areas around the tumor on HBP images;

The quantitative MRI features included:

1. Tumor size: maximum dimension measured on the coronal or axial plane on HBP images;
2. The SIs of tumor and normal liver parenchyma were measured on pre-enhancement, AP, PVP, TP and HBP images respectively, and the following quantitative parameters were calculated:
   1. Tumor to liver contrast ratio (TLR): $\frac{Tumor\_SI \text{post}}{Liver\_SI\text{ }\text{post}}$
   2. Tumor enhancement index (TEI): $\frac{(Tumor\_SI \text{post} \boldsymbol{\div} Liver\_ SI\text{ }\text{post})}{(Tumor\_ SI \text{pre} \div Liver\_SI \text{pre})}$
   3. Relative tumor enhancement (RTE): $\frac{(Tumor\_SI \text{post} - Tumor\_SI \text{pre})}{Tumor\_SI \text{pre}}$
   4. Relative enhancement ratio (RER):$\frac{(Tumor\_SI \text{post} - Tumor\_SI \text{pre})}{(Liver\_SI \text{post} - Liver\_SI \text{pre})}$
3. Reduction rate of T_1_ relaxation time (ΔT_1_%):$\frac{(T\text{1}\text{\_}Pre - T\text{1}\_HBP)}{T\text{1}\_pre}\%$

**2. Supplementary Results**

**Table S2** Univariate analyses of clinical characteristics in the training set.

| Variables | Low Ki-67 LI (n = 35) | High Ki-67 LI (n = 89) | *p* value |
| --- | --- | --- | --- |
| Age (years) | 52 [44, 67] | 54 [54, 69] | 0.420 |
| Sex (male) | 26 (86.7%) | 63 (87.5%) | 0.679 |
| HBsAg |  |  | 0.734 |
| Negative | 3 (10.0%) | 11 (15.3%) |  |
| Positive | 27 (90.0%) | 61 (84.7%) |  |
| ALT (U/L) | 30.60 [16.50, 45.00] | 38.50 [26.75, 68.40] | 0.043* |
| AST (U/L) | 34.50 [26.25, 47.50] | 43.50 [28.50, 49.25] | 0.540 |
| GGT (U/L) | 56.00 [34.50, 124.50] | 73.50 [44.50, 144.25] | 0.558 |
| ALP (U/L) | 78.50 [65.50, 110.50] | 86.50 [66.00, 152.50] | 0.752 |
| ALB (g/L) | 40.50 [36.00, 44.50] | 39.45 [36.70, 42.54] | 0.545 |
| TBIL (µmol/L) | 12.40 [11.30, 17.50] | 14.73 [11.05, 18.62] | 0.599 |
| SCr (U/L) | 75.40 [68.50, 83.92] | 74.50 [66.25, 87.00] | 0.677 |
| PT (s) | 12.24 [11.37, 12.63] | 11.80 [11.40, 12.38] | 0.561 |
| INR |  |  | 0.265 |
| ≤ 1.0 | 10 (33.3%) | 30 (41.7%) |  |
| > 1.0 | 20 (66.7%) | 42 (58.3%) |  |
| NLR | 1.670 [1.04, 2.62] | 2.15 [1.64, 3.64] | 0.036* |
| PLR | 88.51 [53.17, 126.44] | 112.81 [72.54, 163.53] | 0.045* |
| AFP (ng/mL) | 10.25 [3.14, 18.37] | 26.25 [6.61, 96.08] | 0.024* |

Notes: **p*<0.05. Continuous variables are presented as median [inter-quartile range, IQR]. Categorial variables are presented as number (percentage).

Abbreviations: HBsAg, hepatitis B surface antigen; ALT, alanine aminotransferase; AST, aspartate aminotransferase; GGT, glutamyl transpeptidase; ALP, alkaline phosphatase; ALB, albumin; TBIL, total bilirubin; SCr, serum creatinine; PT, prothrombin time; INR, international normalized ratio; NLR, neutrophil to lymphocyte ratio; PLR, platelet to Lymphocyte ratio; AFP, alpha fetoprotein.

**Table S****3** Comparison of semantic MRI findings between low Ki-67 LI and high Ki-67 LI in training set.

| Features | Low Ki-67 LI (n = 35) | High Ki-67 LI (n = 89) | *p* value |
| --- | --- | --- | --- |
| Non-smooth tumor margin | 14 (40.0%) | 70 (78.7%) | <0.001* |
| Hemorrhage | 8 (22.8%) | 50 (56.2%) | 0.031* |
| Necrosis | 10 (28.6%) | 44 (49.4%) | 0.035* |
| Fat component | 11 (31.4%) | 23 (25.8%) | 0.327 |
| Target sign | 7 (20.0%) | 25 (28.1%) | 0.341 |
| Washout | 25 (71.4%) | 64 (71.9%) | 0.714 |
| Rim APHE | 15 (42.9%) | 49 (55.1%) | 0.162 |
| Corona enhancement | 12 (34.3%) | 50 (56.2%) | 0.065 |
| Intratumor arteries | 13 (37.1%) | 28 (31.4%) | 0.434 |
| Radiologic capsule | 25 (71.4%) | 71 (79.7%) | 0.824 |
| Peritumoral hypointensity on HBP | 8 (22.8%) | 33 (37.1%) | 0.171 |

Note: **p*<0.05. Except where indicated, data are numbers of patients, with percentages in parentheses.

Abbreviations: APHE, arterial phase hyperenhancement; HBP, hepatobiliary phase.

**Table S4** Diagnostic of quantitative MRI parameters for predicting Ki-67 LI in the training set.

|  | AUROC (95% CI) | SEN | SPE | Youden | cut-off | *p* value |
| --- | --- | --- | --- | --- | --- | --- |
| Tumor size | 0.640 (0.571 ~ 0.768) | 57.75 | 74.19 | 0.32 | >5.15 cm | 0.007 |
| TLR_TP_ | 0.657 (0.546 ~ 0.739) | 77.46 | 54.84 | 0.32 | ≤0.82 | 0.015 |
| TLR_HBP_ | 0.681 (0.572 ~ 0.789) | 67.61 | 67.74 | 0.35 | ≤0.60 | 0.002 |
| TEI_HBP_ | 0.647 (0.546 ~ 0.755) | 73.25 | 54.84 | 0.32 | ≤0.83 | 0.006 |
| T1_Pre_ | 0.664 (0.534 ~ 0.747) | 77.46 | 54.84 | 0.32 | ＞1328 msec | 0.013 |
| T1_HBP_ | 0.726 (0.632 ~ 0.812) | 73.25 | 71.69 | 0.45 | ＞705 msec | ＜0.001 |

Abbreviations: AUROC, area under the receiver operating characteristic curve; CI, confidence interval; SEN, sensitivity; SPE, specificity; TP, transition phase; HBP, hepatobiliary phase; TLR, tumor to liver contrast ratio; TEI, tumor enhancement index; RTE, relative tumor enhancement; RER, relative enhancement ratio; T1_Pre_: pre-contrast T1 relaxation time; T1_HBP_, T1 relaxation time in the hepatobiliary phase.

**Table S5** ICC results for the quantitative features of the training and validation sets.

|  | Training set | | Validation set | |
| --- | --- | --- | --- | --- |
|  | ICC | 95% CI | ICC | 95% CI |
| Tumor size (cm) | 0.88 | 0.84 ~ 0.92 | 0.86 | 0.82 ~ 0.91 |
| TLR_AP_ | 0.82 | 0.79 ~ 0.86 | 0.84 | 0.80 ~ 0.87 |
| TEI_AP_ | 0.85 | 0.82 ~ 0.89 | 0.81 | 0.78 ~ 0.86 |
| RTE_AP_ | 0.76 | 0.74 ~ 0.80 | 0.78 | 0.74 ~ 0.82 |
| RER_AP_ | 0.80 | 0.78 ~ 0.85 | 0.82 | 0.79 ~ 0.877 |
| TLR_PVP_ | 0.84 | 0.80 ~ 0.89 | 0.85 | 0.80 ~ 0.91 |
| TEI_PVP_ | 0.77 | 0.74 ~ 0.83 | 0.82 | 0.78 ~ 0.86 |
| RTE_PVP_ | 0.84 | 0.80 ~ 0.88 | 0.81 | 0.76 ~ 0.86 |
| RER_PVP_ | 0.76 | 0.72 ~ 0.80 | 0.80 | 0.75 ~ 0.86 |
| TLR_TP_ | 0.83 | 0.78 ~ 0.88 | 0.81 | 0.76 ~ 0.85 |
| TEI_TP_ | 0.82 | 0.78 ~ 0.88 | 0.84 | 0.78 ~ 0.90 |
| RTE_TP_ | 0.80 | 0.78 ~ 0.86 | 0.83 | 0.76 ~ 0.88 |
| RER_TP_ | 0.79 | 0.74 ~ 0.85 | 0.82 | 0.76 ~ 0.86 |
| TLR_HBP_ | 0.86 | 0.82 ~ 0.92 | 0.81 | 0.77 ~ 0.86 |
| TEI_HBP_ | 0.85 | 0.80 ~ 0.91 | 0.89 | 0.84 ~ 0.93 |
| RTE_HBP_ | 0.87 | 0.82 ~ 0.93 | 0.85 | 0.81 ~ 0.91 |
| RER_HBP_ | 0.81 | 0.78 ~ 0.86 | 0.84 | 0.79 ~ 0.90 |
| T1_Pre_ (msec) | 0.83 | 0.78 ~ 0.88 | 0.87 | 0.82 ~ 0.93 |
| T1_HBP_ (msec) | 0.82 | 0.79 ~ 0.86 | 0.86 | 0.81 ~ 0.92 |
| ΔT1% | 0.78 | 0.74 ~ 0.83 | 0.80 | 0.76 ~ 0.85 |

Abbreviations: AP, arterial phase; PVP, portal venous phase; TP, transition phase; HBP, hepatobiliary Phase; TLR, tumor to liver contrast ratio; TEI, tumor enhancement index; RTE, relative tumor enhancement; RER, relative enhancement ratio; T1_Pre_: pre-contrast T1 relaxation time; T1_HBP_, T1 relaxation time in the hepatobiliary phase; ΔT1%, reduction rate of T1 relaxation time; ICC, intraclass correlation coefficient.

**Figure S1** The DCAs for the clinical, imaging and combined models of the training set.


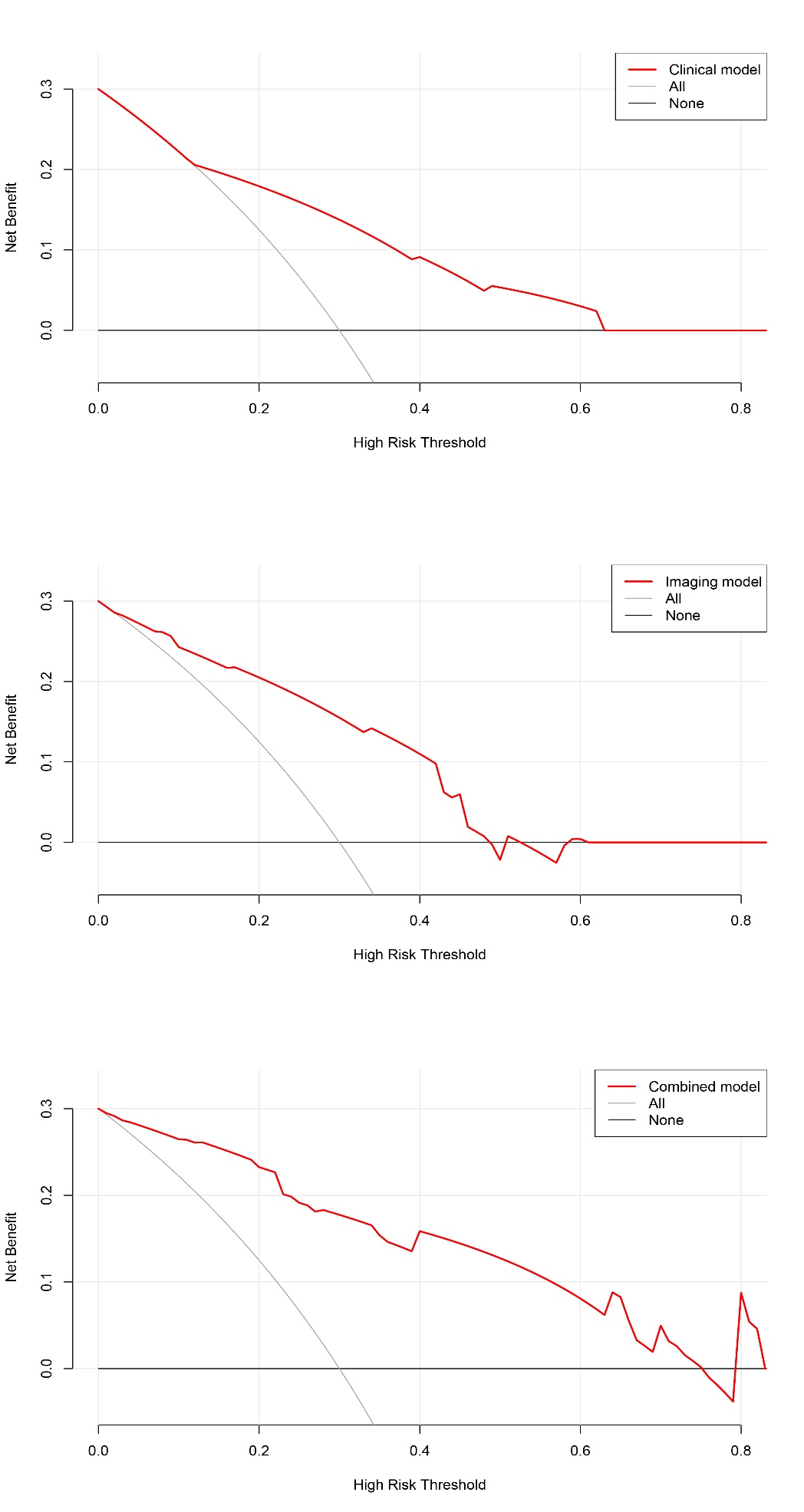

Supplement: Supplementary file 1 [file DataSheet_1.docx]
